# ‘*Candidatus* Tisiphia’ is a widespread Rickettsiaceae symbiont in the mosquito *Anopheles plumbeus* (Diptera: Culicidae)

**DOI:** 10.1111/1462-2920.16486

**Published:** 2023-09-02

**Authors:** Helen R. Davison, Jessica Crozier, Stacy Pirro, Helge Kampen, Doreen Werner, Gregory D. D. Hurst

**Affiliations:** ^1^ Institute of Infection, Veterinary and Ecological Sciences (IVES) University of Liverpool Liverpool UK; ^2^ Iridian Genomes Bethesda Maryland USA; ^3^ Institute of Infectology (IMED) Friedrich‐Loeffler‐Institut, Federal Research Institute for Animal Health Greifswald Isle of Riems Germany; ^4^ Land Use and Governance Leibniz Centre for Agricultural Landscape Research (ZALF) Müncheberg Germany

## Abstract

Symbiotic bacteria can alter host biology by providing protection from natural enemies, or alter reproduction or vectoral competence. Symbiont‐linked control of vector‐borne disease in *Anopheles* has been hampered by a lack of symbioses that can establish stable vertical transmission in the host. Previous screening found the symbiont ‘*Candidatus* Tisiphia’ in *Anopheles plumbeus*, an aggressive biter and potential secondary vector of malaria parasites and West Nile virus. We screened samples collected over 10‐years across Germany and used climate databases to assess environmental influence on incidence. We observed a 95% infection rate, and that the frequency of infection did not fluctuate with broad environmental factors. Maternal inheritance is indicated by presence in the ovaries through FISH microscopy. Finally, we assembled a high‐quality 1.6 Mbp draft genome of ‘*Ca*. Tisiphia’ to explore its phylogeny and potential metabolic competence. The infection is closely related to strains found in *Culicoides* biting midges and shows similar patterns of metabolism, providing no evidence of the capacity to synthesize B‐vitamins. This infection offers avenues for onward research in anopheline mosquito symbioses. Additionally, it provides future opportunity to study the impact of ‘*Ca*. Tisiphia’ on natural and transinfected hosts, especially in relation to reproductive fitness and vectorial competence and capacity.

## Graphical abstract

Control of mosquito populations and their vector competence is commonly achieved using symbionts, but efforts are hampered in anophelines by limited compatible symbiotic partners. We establish that the Rickettsiaceae symbiont, *Ca*. Tisiphia', resides in the ovaries of *Anopheles plumbeus* and infects >95% of German populations. We assemble the symbiont's genome and provide supporting phylogenomic and metabolic analyses. This infection provides an opportunity onward research in anopheline mosquito symbioses, and the control of vector‐borne disease.

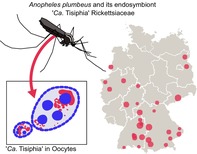

## INTRODUCTION

Bacterial symbionts in insects form vital components of their host's biology, ecology and evolution. They are known to influence how insects reproduce, how they respond to environmental stress and their susceptibility to pathogen attack (Dunbar et al., 2007; Hayashi et al., 2016; Hendry et al., 2014; Himler et al., 2011; Vega et al., 2012; Xie et al., 2014). In many cases, symbionts are vertically inherited from one generation to the next, usually through the maternal germline, and may become intrinsically linked with their host physiology, metabolism and development (Buchner, 1965; Giorgini et al., 2010; Kremer et al., 2009; Moran et al., 2008; Zchori‐Fein et al., 2006). Most importantly, symbionts have been deployed in the control of vector populations and vector competence (Hoffmann et al., 2011; Laven, 1967).

Success in symbiont‐mediated disease control has been restricted to species from the genera *Aedes* and *Culex*. Transinfection with *Wolbachia* from a drosophilid fly has been successfully used to alter vector competence and lower risk of catching dengue fever from *Aedes aegypti* L. in endemic areas (Hoffmann et al., 2011; Pereira et al., 2018; Walker et al., 2011). Similarly, *Culex pipiens fatigans* L. has a natural infection with a *Wolbachia* (*w*Pip) that causes cytoplasmic incompatibility (Laven, 1967), and has been used to transinfect *Culex quinquefasciatus* Say, 1823 as a potential method of population size control (Atyame et al., 2015). However, important vector species within the genus *Anopheles* are poorly receptive to transinfection with *Wolbachia* (Hughes et al., 2014) and are rarely naturally infected (Kittayapong et al., 2000; Ricci et al., 2002), with a single well‐established case of natural *Wolbachia* infection (Walker et al., 2021). Therefore, it is desirable to identify additional symbionts compatible with anopheline mosquitoes that are either more capable of surviving transinfection or alter vector competence in the native host.

We previously detected the symbiont ‘*Candidatus* Tisiphia’ (= Torix group *Rickettsia*) in three *Anopheles plumbeus* Stephens, 1828 individuals sampled in the United Kingdom (Pilgrim et al., 2021). *An. plumbeus* is broadly distributed across Europe and is an indiscriminate biter. This species is also capable of transmitting West Nile virus and malaria parasites, although these pathogens do not natively occur in the majority of the mosquito species' known distribution range, and vector competence has only been tested in the laboratory setting (Bueno‐Marí & Jiménez‐Peydró, 2011; Dekoninck et al., 2011; Schaffner et al., 2012). It has been highlighted as a species that could act as a secondary vector of ‘tropical’ disease agents as changing climate causes their northward spread and their associated primary hosts like *Aedes albopictus* Skuse, 1894 (Heym et al., 2017; Schaffner et al., 2012).

‘*Ca*. Tisiphia’ appears to be particularly associated with hosts deriving from wet or aquatic environments and may originate from symbionts of freshwater ciliates (Driscoll et al., 2013; Kang et al., 2014; Schrallhammer et al., 2013). Infection with this symbiont occurs in a broad range of invertebrates, from annelids to gastropods to arthropods (Pilgrim et al., 2021), as well as in algae (Hollants et al., 2013) and amoebae (Dyková et al., 2013). Their relatives in the genus *Rickettsia* are capable of nutritional symbioses, protecting against fungal infections and reproductive manipulation (Bodnar et al., 2018; Giorgini et al., 2010; Hendry et al., 2014; Hurst et al., 1994). However, the known effects of ‘*Ca*. Tisiphia’ are limited to an association with increased host size in *Torix* leeches, and weak impacts on fecundity in the bedbug, *Cimex lectularius* L. (Kikuchi & Fukatsu, 2005; Thongprem et al., 2020). There is no observed congruence of host and symbiont phylogeny, indicating that host shifts occur commonly and that long‐standing associations with species are rare (Davison et al., 2022). External influence such as temperature or natural enemies can influence the prevalence of symbionts in host populations, but the importance of these factors are not yet established for ‘*Ca*. Tisiphia’ (Cass et al., 2016; Corbin et al., 2017; Leclair et al., 2017).

In this study, we used PCR assays to establish the extent of ‘*Ca*. Tisiphia’ infection in *An. plumbeus* mosquitoes collected throughout Germany in collaboration with a citizen science initiative. We provide evidence that the symbiont is vertically transmitted through the maternal germline through fluorescence in situ hybridisation (FISH) imaging of ovary material. The symbiont genome was sequenced and assembled and then examined through bioinformatics approaches to establish potential nutritional or protective symbioses. We also assessed associations with temperature, precipitation and forest type.

## EXPERIMENTAL PROCEDURES

### Mosquito sampling

Materials for screening ‘*Ca*. Tisiphia’ infection in *An. plumbeus* were collected from 2012 to 2021 across Germany by part of the authors and citizen volunteers of the mosquito atlas (Mückenatlas) project (Werner et al., 2014). The samples comprised 245 females and 10 males, with the low number of males reflecting collection methods favouring biting females rather than an intrinsic sex ratio bias. Specimens were stored in 70% ethanol or dry (see Supporting Information for storage and exact geographic information). Post hoc analysis indicated storage method did not affect detection of symbionts by PCR assay.

Additional live specimens were also collected in Germany as larvae in 2021 and raised to adults in water collected from their larval pools for the purpose of imaging and genomic sequencing. These specimens were either killed by flash freezing in liquid nitrogen prior to genomic DNA extraction, or in 4% paraformaldehyde solution prior to fluorescence imaging.

### DNA extraction and PCR screening of *An. plumbeus* for ‘*Ca*. Tisiphia’

Promega Wizard® Genomic DNA Purification kit (Promega, USA) was used for DNA preparation as per manufacturer instructions. The presence and quality of DNA was checked with a combination of HCO/C1J primers HCO_2198 (5'‐TAA ACT TCA GGG TGA CCA AAA AAT CA‐3')/CIJ_1718 (5'‐GGA GGA TTT GGA AAT TGA TTA GT‐3') (Folmer et al., 1994; Hajibabaei et al., 2005; Siozios et al., 2020); the presence of strong bands indicated that DNA was intact and that the positive results of subsequent PCR could be trusted. ‘*Ca*. Tisiphia’ presence was assessed with a PCR assay amplifying the 320‐bp region of the 17 kDa outer‐membrane protein (OMP) gene with primer pair Ri17kD_F (5'‐TCTGGCATGAATAAACAAGG‐3')/Ri17kD_R (5'‐ACTCACGACAATATTGCCC‐3'; Pilgrim et al., 2017). PCR conditions used were as follows: 95°C for 5 min, followed by 35 cycles of denaturation (94°C, 30 s), annealing (54°C, 30 s) and extension (72°C, 120 s).

A selection of ‘*Ca*. Tisiphia’ amplicons from collections across time and space were Sanger‐sequenced through Eurofins Genomics Europe barcoding service (Ebersberg, Germany) and identity was confirmed by comparing the sequences to the NCBI database via BLAST homology searches. Confirmed sequences have subsequently been deposited in the same database under accession numbers OQ512853‐OQ512860.

### De novo sequencing, assembly and annotation

A combination of short and long read sequencing was used to construct scaffolds of the ‘*Ca*. Tisiphia’ genome. For short reads, Iridian Genomes (Bethesda, USA) extracted and processed DNA of a single laboratory‐reared *An. plumbeus* male for Illumina sequencing deposited under bioproject accession PRJNA694375. The short reads of *An. plumbeus* were cleaned with Trimmomatic v0.36 (Bolger et al., 2014) and quality‐checked with FASTQC v0.11.9 (Babraham Bioinformatics, 2019). For long reads, genomic DNA from one male was extracted with the QIAGEN Genomic‐tip 20/G kit (QIAGEN, Netherlands) as per manufacturer instructions for ultra‐low PacBio sequencing carried out by the Centre for Genomic Research, University of Liverpool, UK. Long‐read sequences have been deposited under bioproject accession number PRJNA901697.

A combination of long‐ and short‐read data were used to assemble as complete a genome for ‘*Ca*. Tisiphia’ as possible. First, the ‘*Ca*. Tisiphia’ genome was identified in the Illumina short reads and assembled through Minimap2, MEGAHIT and MetaBAT2 as per the pipeline that can be found in the github repository in Davison (2022) and described in Davison et al. (2022). Second, PacBio HiFi long read sequences were assembled using Flye v2.9. 1‐b1780 with the ‘‐meta’ flag to improve sensitivity for low coverage reads. Third, the long‐read assembly was queried against a local blast database of *Rickettsia* and ‘*Ca*. Tisiphia’ genomes (including the Illumina assembly from the first step) to identify sequences belonging to this strain of ‘*Ca*. Tisiphia’. Finally, the long‐read assembly was polished with the Illumina reads using Polypolish v0.5.0 (Wick & Holt, 2022) with default settings, giving 23 final scaffolds.

### Phylogeny and metabolic predictions

Annotation of the final assembly was carried out with InterProScan v5 (Jones et al., 2014). Prediction of metabolic pathway presence and completion was carried out through Anvi'o v7 using KEGG kofams and COG20 (Aramaki et al., 2020; Eren et al., 2021; Galperin et al., 2021). NRPS pathways were investigated with AntiSMASH 6.0 (Blin et al., 2021).

The genome of the *An. plumbeus* ‘*Ca*. Tisiphia’ strain was compared to other existing ‘*Ca*. Tisiphia’ genomes through Anvi'o 7. The new genome will subsequently be referred to as TsAplum, a contraction of ‘*Ca*. Tisiphia’ and its host, *An. plumbeus*. A core genome consisting of 205 gene clusters that contain a total of 3690 genes was estimated through Anvio‐7. Summaries of gene clusters can be found in Table S5 Phylogenies were estimated from single copy gene clusters with IQTREE v2.2.0.3 using Model Finder Plus and with 1000 ultrafast bootstraps (Hoang et al., 2018; Kalyaanamoorthy et al., 2017; Minh et al., 2020). The model selected by Model Finder Plus is Q.plant + F + R4. A supporting phylogeny to confirm the identity of 17 kDa OMP genes was produced with the model TIM2 + F.

### Fluorescence in situ microscopy

Reproductive organs of a single *An. plumbeus* female and a single male were dissected and incubated in cold 4% paraformaldehyde for 3 h, agitated gently every 30 min, then washed with cold PBS for 5 min two times. Tissue was stained with Hoechst 33342 (Thermo Fisher Scientific, USA) for 30 min at room temperature, then hybridised overnight at room temperature with hybridisation buffer (5× SSC, 0.01% SDS, 30% formamide) and 5′‐CCATCATCCCCTACTACA‐(ATTO 633)‐3′ oligonucleotide probe specific to ‘*Ca*. Tisiphia’ 16S rRNA (as described in Pilgrim et al., 2017 and Thongprem et al., 2020). Hybridised tissue was washed in wash buffer (5× SSC, 0.01% SDS) at 48°C for 60 min with gentle shaking every 20 min. Samples were then mounted in Vectashield (Vector Laboratories, Inc., USA). Images were taken with a ZEISS LSM 880 confocal microscope through ZEISS Zen black, and final images were annotated in Inkscape v1.2 (Inkscape Project, 2020).

### Association of symbiont prevalence with geographic and climatic information

Annual average monthly temperature and precipitation data were retrieved for each sample's coordinate and year from TerraClim (Abatzoglou et al., 2018), which has a spatial resolution of ~4 km (1/24th degree). Forest cover data were retrieved from Copernicus land datasets for 2018 (European Union, 2018) and raster data for forest type extracted in QGIS 3.16 (QGIS.org, 2020) within a 3‐km radius of each sample location. *An. plumbeus* has historically been recorded to have a maximum flight range of up to 13 km (Becker et al., 2010). However, this is based on one single study from 1925 and is not verified by other sources. As such, we chose an estimated range of 3 km based on the average flight ranges other anopheline mosquito species (Becker et al., 2010; Verdonschot & Besse‐Lototskaya, 2014). Scikitlearn's standard scaler (Pedregosa et al., 2011) was applied to the data before performing a generalised linear model with a binomial logit link function on data with the following formula: ‘Infected’ denotes if a sample was PCR positive for ‘*Ca*. Tisiphia’, ‘tasmin’ is the annual average minimum temperature, ‘tasmax’ is the annual average maximum temperature, ‘precip’ is the annual average precipitation, ‘forest_ratio’ is the ratio of deciduous to coniferous forest.

Infected~tasmin+tasmax+precip+forest_ratio

All statistics and geographic inferences were carried out in Python with the packages Statsmodel and Scikit‐learn (Pedregosa et al., 2011; Rossum & Drake, 2009; Seabold & Perktold, 2010). QGIS 3.16 was used to produce maps and extract raster data for forest types before passing it to Python for analysis (QGIS.org, 2020). All other figures were produced with Matplotlib and Seaborn (Hunter, 2007; Waskom & Seaborn Development Team, 2020).

## RESULTS AND DISCUSSION

### Distribution and predicted environment

**FIGURE 1:**
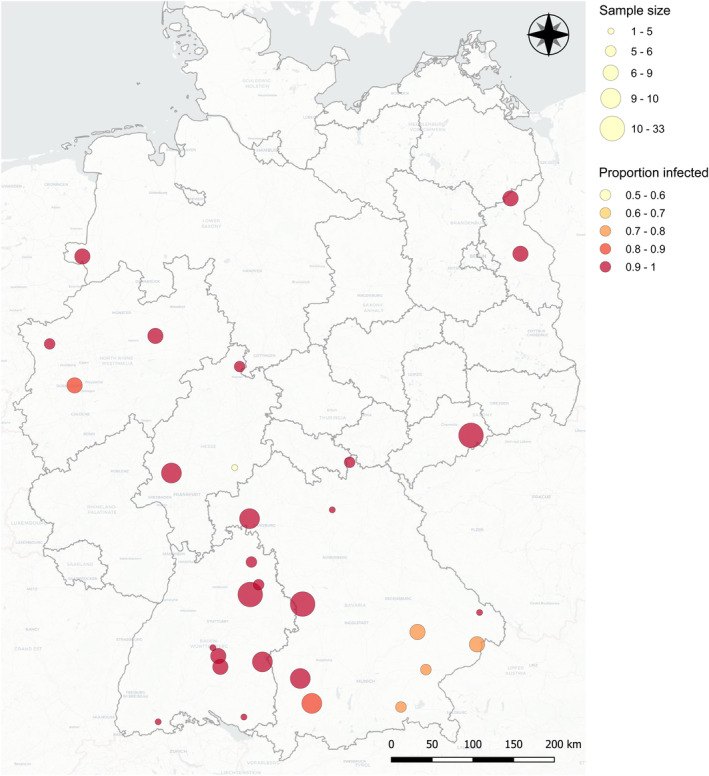
A map of ‘*Ca*. Tisiphia’ infection rates in *Anopheles plumbeus* across Germany with the size of the circle representing the number of individuals sampled and the colour indicating the proportion of ‘*Ca*. Tisiphia’ infected individuals. Red = 90%–100% infection to light yellow = 50%–60% infection. Source data can be found in Table S1.

PCR assay of ‘*Ca*. Tisiphia’ found 244 of 255 (95.6%) *An. plumbeus* samples to be positive across all sites examined in Germany. The few negative specimens were found mostly in the southeast of the country (Figure 1 and Figure S1). There was no difference in infection rate between male and female samples. We can be confident prevalence is very high (binomial confidence intervals for prevalence if PCR has no false negatives/positives: 91%–97%); potential false negatives from PCR failure of old template DNA make it possible infection is fixed or nearly fixed; 17 kDa PCR amplicons were sequenced for five specimens, and these proved to be identical in sequence, indicating a single circulating strain. The infection seems to be stable in frequency, with samples from all years spanning 2012 to 2022 displaying similar rates of infection (Figures S1 and S2, Table S1). There is also no clear evidence of an association between ‘*Ca*. Tisiphia’ infection rates in *An. plumbeus* and average minimum or maximum temperature, precipitation or forest types (described in Supporting Information Figures and Results).

### Phylogeny and metabolism

**FIGURE 2:**
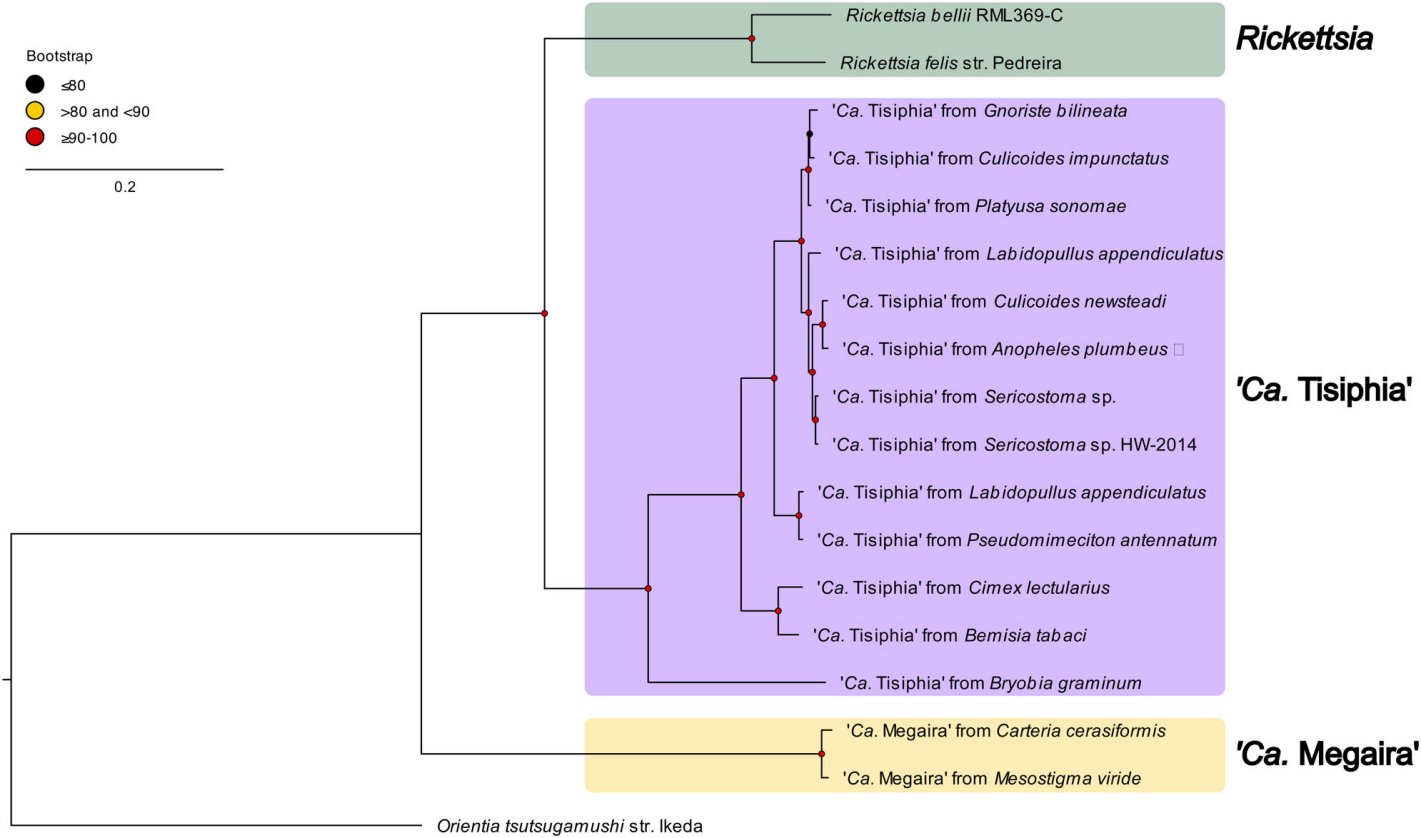
Genome‐wide phylogeny of ‘*Ca*. Tisiphia’, *Rickettsia* and ‘*Ca*. Megaira’. Maximum likelihood (ML) phylogeny constructed from 205 single‐copy gene clusters that contain a total of 3690 genes (summarised in Table S5). The new genome is indicated by ◄, and bootstrap values based on 1000 replicates are indicated with coloured circles (red, ≥90 and <100; yellow, >80 and ≤90; black, ≤80). Accession numbers for each genome are available in Table S2.

The symbiont genome assembled into 1.6 Mbp across 31 contigs, with high CheckM completeness (94.55% vs. 97.63% for the closed ‘*Ca*. Tisiphia’ genome of *Culicoides impunctatus*) (Table 1). The small genome size of 1.6 Mbp is typical of *Rickettsia*‐like bacteria which vary from 1.1 to 2.3 Mbp (Davison et al., 2022; Diop et al., 2018). TsAplum has a single full set of rRNAs (16S, 5S and 23S), and GC content is ~33%. It also has several genes with repeat domains that are enable protein–protein interactions and which are prevalent in *Wolbachia* and other symbionts (Davison et al., 2023; Rice et al., 2017; Siozios et al., 2013). Core gene analysis indicated the bacteria sequenced from *An. plumbeus* are most closely related to a ‘*Ca*. Tisiphia’ found in the biting midge *Culicoides newsteadi* (Figure 2).

**TABLE 1:** Summary of the genome assembly for '*Ca*. Tisiphia' str. TsAplum.

Strain name	TsAplum
Symbiont genome accession	SAMN31737641
Host	*Anopheles plumbeus*
Raw reads accession	Pacbio SRR22298143, Illumina SRR13516402
Total nucleotides (bp)	1,622,210
Contigs	31
Completeness (CheckM)	94.55%
Contamination (CheckM)	1.66%
GC content	32.82%
N50	62,798
Number of CDS	1701
Avg. CDS length (bp)	788
Coding density	82.57%
rRNAs	1 × 5S, 1 × 16S, 1 × 23S
tRNAs	31
ORFs with Ankyrin repeat domains	3
ORFs with Leucine rich repeats	1
ORFs with Tetratricopeptide repeats	6

All infections tested were the same strain of ‘*Ca*. Tisiphia’ (Figure S5). General features of both genomes are consistent with other ‘*Ca*. Tisiphia’ (Table 1 and Tables S2–S4).

Overall, the predicted metabolic capabilities of the ‘*Ca*. Tisiphia’ bacteria found in *An. plumbeus* mirrors that of other members of its genus (Figures S6 and S7, and Table S4). Metabolic pathway that would contribute to nutritional symbiosis such as B vitamin production were not predicted, nor was there any evidence for a NRPS/PKS system for small molecule synthesis that might be associated with protection (Tables S3 and S4). The genome does have several predicted toxin/anti‐toxin systems as well as Tat, Sec, VirB (Type IV) secretion pathways, all of which are essential in various symbiont‐host interactions (Dale & Moran, 2006; Masui et al., 2000; Meloni et al., 2003; Tseng et al., 2009; Wu et al., 2004). Additionally, this ‘*Ca*. Tisiphia’ strain has a number of ORFs containing ankyrin‐ and leucine‐rich repeats, which are thought to be important in interactions with cognate eukaryotic proteins (Rice et al., 2017; Siozios et al., 2013). Thus, the genome itself, whilst firmly placing the symbiont in the context of the genus, identifying relatedness to other strains and consistent with its symbiotic nature, does not raise obvious hypotheses about the impact of infection on the host. Phenotype studies are required to properly assess the influence of this strain and its relatives on its various hosts. Key studies would address the factors driving the spread of the symbiont into the population (testing for beneficial aspects of infection, cytoplasmic incompatibility and paternal inheritance) and impacts on viral infection and transmission outcomes.

### FISH imaging

**FIGURE 3:**
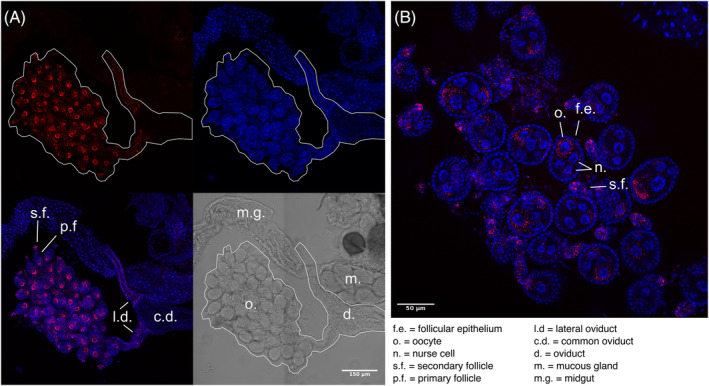
Fluorescence in situ microscopy images of *Anopheles plumbeus* ovaries infected with ‘*Ca*. Tisiphia’. Red shows ‘*Ca*. Tisiphia’ stained with ATTO‐633, blue are host nuclei stained with Hoechst 33342. Panels show (A) the whole female reproductive organ outlined in white and a breakdown of each light channel and (B) a close up of the ovaries where infection is localised within the primary and secondary follicles. White bars indicate (A) 150 micrometres and (B) 50 micrometres.

*‘Ca*. Tisiphia’ infection is observed in oocytes and oviduct branches but is not detected in testes, indicating that maternal inheritance is the method of transmission, and that paternal inheritance is unlikely (Figure 3 vs. Figure S8). Localisation and clear polarity of the infection in ovaries strongly suggest that this is a maternally inherited infection (Figure 3). The bacteria cluster around the oocyte of the primary follicles as well as in the lateral ducts and secondary follicles. In *Drosophila melanogaster*, *Wolbachia* is similarly polarised to one end of the primary follicles to the oocyte (Ferree et al., 2005), and *Sodalis* endosymbionts in *Proechinophthirus fluctus* appear to use lateral oviducts to access the ovaries (Boyd et al., 2016).

### Final conclusions

*An. plumbeus* and its infection with the bacterium ‘*Ca*. Tisiphia’ make a good model for symbioses in *Anopheles* mosquitoes, as well as for ‘*Ca*. Tisiphia’ infections more generally. Outside of *An. plumbeus*, there is only a single well‐substantiated case of *Wolbachia* in anopheline mosquitoes (Walker et al., 2021) and no reports of other heritable microbes. The symbiosis with *An. plumbeus* is clearly evidenced, likely heritable and occurs in a species that has previously been successfully bred in a laboratory colony (Kotter, 2005). Beyond this, *An. plumbeus* is a species of interest with the ability to transmit West Nile virus and *Plasmodium* parasites (Dekoninck et al., 2011; Schaffner et al., 2012). ‘*Ca*. Tisiphia’ infection in *An. plumbeus* provides a viable avenue for symbiont‐mediated vector modification and control that can be tested in anopheline species. It is also an example of a temporally and spatially stable infection of non‐pathogenic *Rickettsiaceae* and a good comparison to fluctuating systems like Belli *Rickettsia* in *Bemisia tabaci* (Bockoven et al., 2020).

Symbiosis may be beneficial or deleterious to the host, and indeed this status vary according to biotic and abiotic pressures (Drew et al., 2021). Future work should establish the effects of ‘*Ca*. Tisiphia’ in the *An. plumbeus* and how it is maintained. There are three non‐mutually exclusive methods by which purely maternally inherited symbionts might be maintained in their host:sex ratio distortion, cytoplasmic incompatibility or beneficial contribution to female host survival and reproduction. Sex ratio distortion is unlikely because the infection is present in both sexes and at rates of >95% of the wild population. Cytoplasmic incompatibility should be tested as it is observed in a wide variety of symbiotic bacteria (*Wolbachia, Cardinium, Rickettsia, Spiroplasma*) (Altinli et al., 2018; Gillespie et al., 2018; Gotoh et al., 2007; Hayashi et al., 2016; Pollmann et al., 2022; Shropshire et al., 2020; Walker et al., 2011). Cytoplasmic incompatibility would be particularly significant as it can be used in disease vector control (Hoffmann et al., 2011; Laven, 1967). Beneficial contributions by symbionts can be diverse and include nutritional and protective roles, both of which may be key features of host biology and can result in closely linked evolution, as seen with symbionts like *Buchnera* and *Hamiltonella* in aphids (Buchner, 1965; Leclair et al., 2017) or *Stammera* in shield beetles (Salem et al., 2020).

Investigations of how transinfection in alternative hosts affect the symbiont's function will also be desirable. Symbionts like *Wolbachia* are known to produce functionally interesting phenotypes related to vector competence when transferred from the original host into other, naïve species (Moreira et al., 2009). Alongside this, impacts on host function and physiology, and potential means of spread into natural populations would need to be assessed. A first step to establishing transinfection would be to isolate ‘*Ca*. Tisiphia’ into cell culture, which would also represent an important community resource for onward studies.

In summary, ‘*Ca*. Tisiphia’ is found in approximately 95% of *An. plumbeus* individuals from Germany and forms a well‐established, stable, and heritable infection that persists across space and time. Metabolic potential is typical of similar symbiotic bacteria species, and we find no evidence of large‐scale environmental factors influencing rates of infection. However, ‘*Ca*. Tisiphia’ and *An. plumbeus* provide a unique opportunity to study the effects of a native symbiont infection in anopheline mosquitoes, as well as explore its potential use for disease mitigation in other species that cannot be infected with currently used symbionts.

## AUTHOR CONTRIBUTIONS

**Helen R. Davison:** Conceptualization (equal); data curation (lead); formal analysis (lead); funding acquisition (supporting); investigation (lead); methodology (lead); resources (equal); supervision (equal); visualization (lead); writing – original draft (lead); writing – review and editing (lead). **Jessica Crozier:** Investigation (equal); writing – review and editing (equal). **Stacy Pirro:** Investigation (supporting); resources (equal); writing – review and editing (equal). **Helge Kampen:** Investigation (supporting); methodology (supporting); resources (equal); writing – review and editing (equal). **Doreen Werner:** Investigation (supporting); methodology (supporting); resources (equal); writing – review and editing (equal). **Gregory D. D. Hurst:** Conceptualization (equal); funding acquisition (lead); methodology (equal); project administration (equal); supervision (equal); writing – original draft (supporting); writing – review and editing (equal).

## CONFLICT OF INTEREST STATEMENT

The authors declare no conflicts of interest.

## ETHICS STATEMENT

All samples were collected in accordance with ethical standards for insect collection.

## Supporting information


**Data S1:** Supporting Data.

**Data S2:** Supporting Information.

## Data Availability

Data available in article supplementary material and in Genome bioproject accessions: PRJNA694375 and PRJNA901697. PCR sequences are deposited in accessions OQ512853‐OQ512860.
