# Supplementary material for: ‘Candidatus Tisiphia’ is a widespread Rickettsiaceae symbiont in the mosquito Anopheles plumbeus (Diptera: Culicidae)
Source: Environ Microbiol. 2023 Sep 2;25(12):3064–74. doi: 10.1111/1462-2920.16486 (PMC10947512; doi:10.1111/1462-2920.16486)
Supplement: Supplementary file 2 — Data S2: Supporting Information. [file EMI-25-3064-s001.pdf]

Supplementary figures and methods for: ‘*Candidatus* Tisiphia’ is a widespread Rickettsiaceae symbiont in the mosquito *Anopheles plumbeus* (Diptera: Culicidae)

Authors

Helen R. Davison[1], Jessica Crozier[1], Stacy Pirro[2], Helge Kampen[3], Doreen Werner[4], Gregory D.D. Hurst[1]

Affiliations

1. Institute of Infection, Veterinary and Ecological Sciences, University of Liverpool, Crown Street Liverpool L69 7ZB UK
2. Iridian Genomes, Bethesda, MD, USA
3. Friedrich-Loeffler-Institut, Federal Research Institute for Animal Health, 17493 Greifswald – Isle of Riems
4. Leibniz Centre for Agricultural Landscape Research (ZALF), 15374 Müncheberg, Germany

Corresponding Author

Helen R. Davison - davstaff@liverpool.ac.uk

Data availability statement

Genome bioproject accessions: PRJNA694375 and PRJNA901697. PCR sequences are deposited in accessions OQ512853-OQ512860

Environmental data analysis results

There is no clear evidence of an influence on '*Ca*. Tisiphia' infection rates in *An. plumbeus* caused by average minimum or maximum temperature, precipitation, or forest types (Supplementary Figure 2, 3, and 4). While there appears to be a significant effect of precipitation on the number of uninfected individuals, this could be an artifact of increased water availability leading to more mosquitoes and thus a higher chance of detecting rarer uninfected individuals (Supplementary Figure 2). No variation is unsurprising as it appears to be a very high prevalence infection. We also acknowledge that using climate databases to retroactively find data is not as accurate as field measurements. However, results agree with previous field observations of *Rickettsia* infection in *Acyrthosiphon pisum* in Japan where distribution of symbiont infection did not correlate with precipitation or temperature (Tsuchida *et al.*, 2002). We chose to use the high resolution TerraClim database, but this may still mask small differences in microenvironments are limited to mostly abiotic data. We encourage future symbiosis research to consider environmental measurements to describe the ecology of these organisms more comprehensively.

Generalized Linear Model Regression Results

**Dep. Variable:**	Infected	**No. Observations:**	255
**Model:**	GLM	**Df Residuals:**	250
**Model Family:**	Binomial	**Df Model:**	4
**LinkFunction:**	Logit	**Scale:**	1
**Method:**	IRLS	**Log-Likelihood:**	-38.037
**Date:**	Tue, 6 Jun 2023	**Deviance:**	76.075
**Time:**	16:13:14	**Pearson chi2:**	464
**No. Iterations:**	7	**Pseudo R-squ. (CS):**	0.05564
**Covariance Type:**	nonrobust		

	**coef**	**std err**	**z**	**P>|z|**	**[0.025**	**0.975]**
**Intercept**	6.2208	1.417	4.389	0	3.443	8.999
**tasmin**	-0.699	1.301	-0.537	0.591	-3.248	1.85
**tasmax**	-2.1218	1.593	-1.332	0.183	-5.244	1
**precip**	-3.0896	1.41	-2.192	0.028	-5.853	-0.326
**forest_ratio**	1.0468	2.075	0.504	0.614	-3.021	5.114

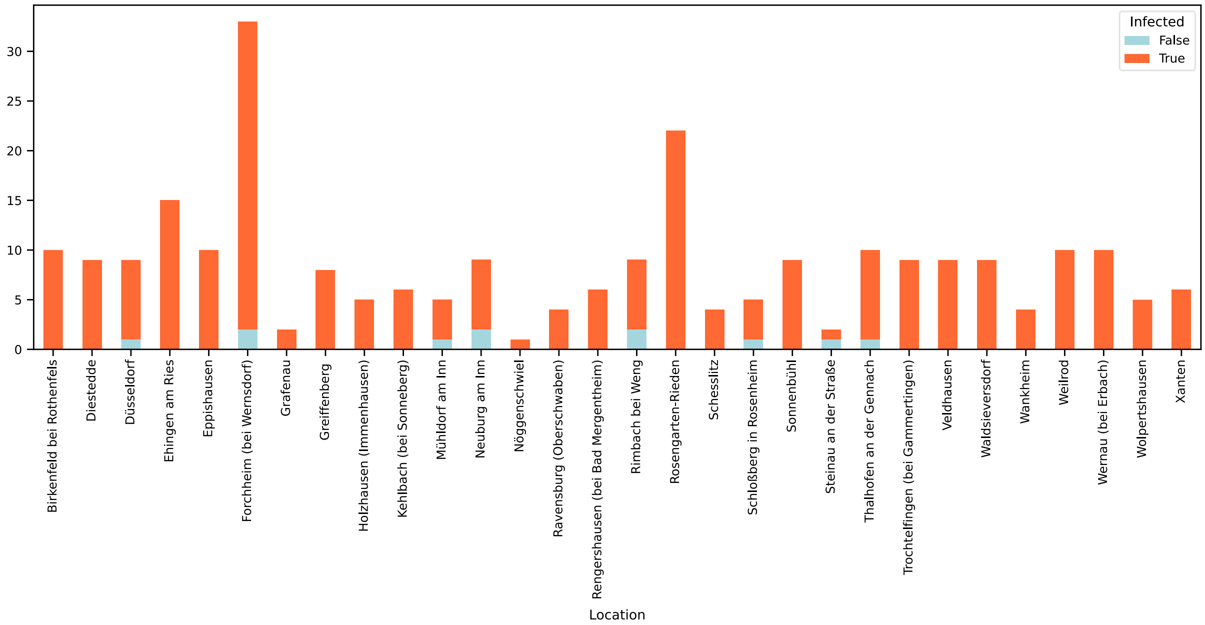

Supplementary Figure 1. '*Ca*. Tisiphia' infection rates by site'. Infected samples are shown in orange, uninfected are shown in light blue. Source data can be found in Supplementary Table S1.

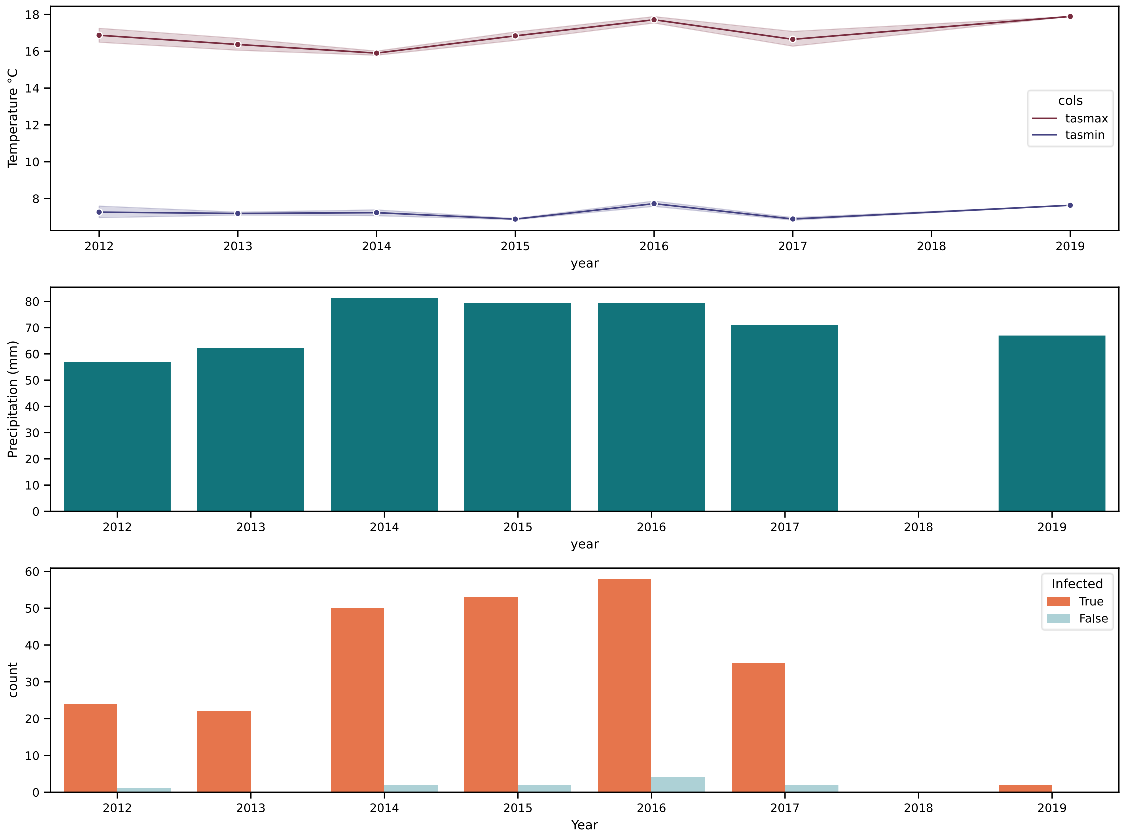

Supplementary Figure 2. Environmental data for *Anopheles plumbeus* collection sites across Germany extracted from the TerraClim database. (Top) average annual minimum and maximum temperature across all *An. plumbeus* collection sites in Germany. (Middle) average annual precipitation across all sites. (Bottom) counts of infected and uninfected individuals across all sites where light blue = uninfected and orange = infected.

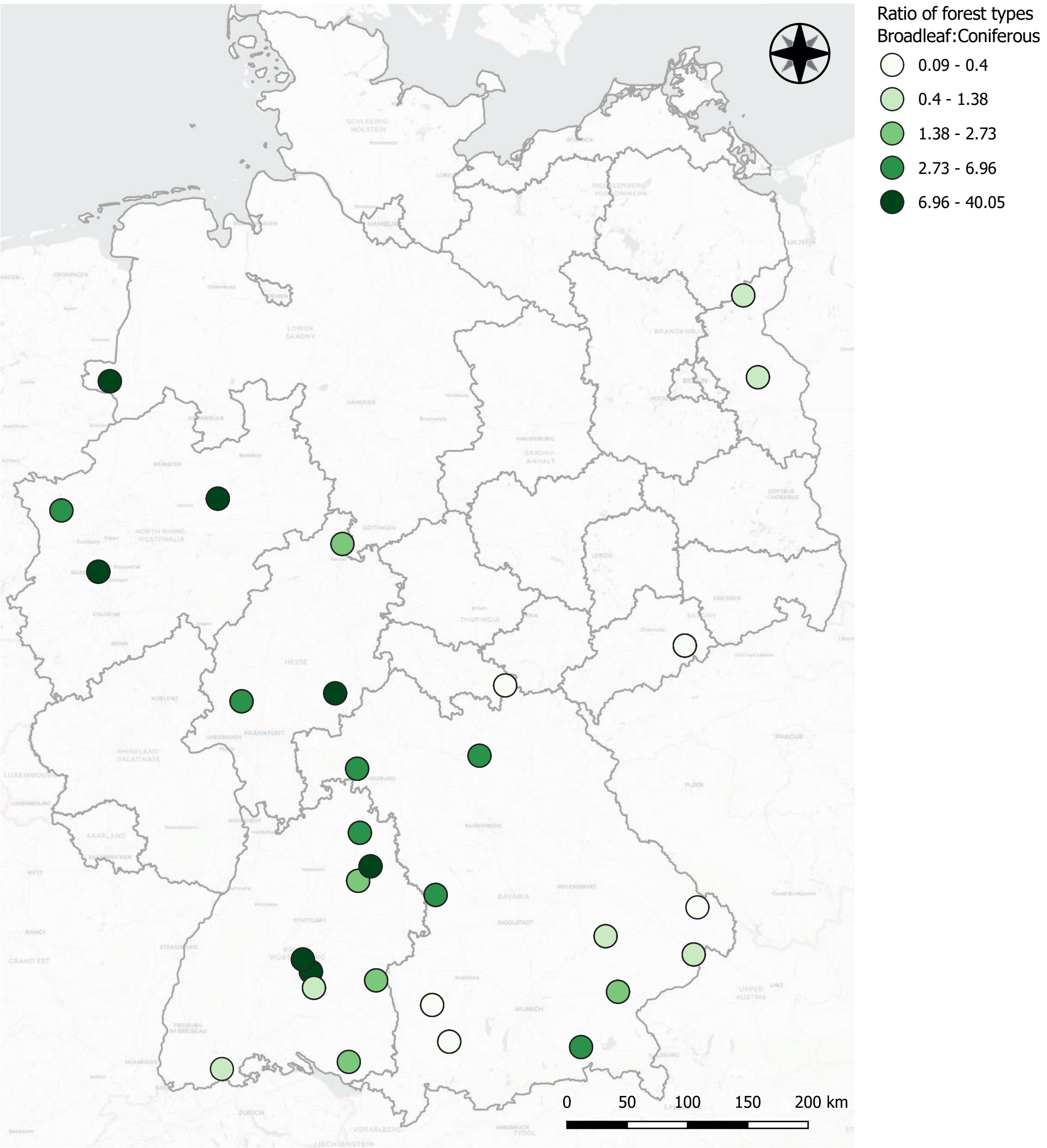

Supplementary Figure 3. The ratio of broadleaf to coniferous forest in a 3km radius of each collection site. Darker green indicates more broadleaf, lighter green indicates closer to equal proportions. Source data can be found in Supplementary Table S1.

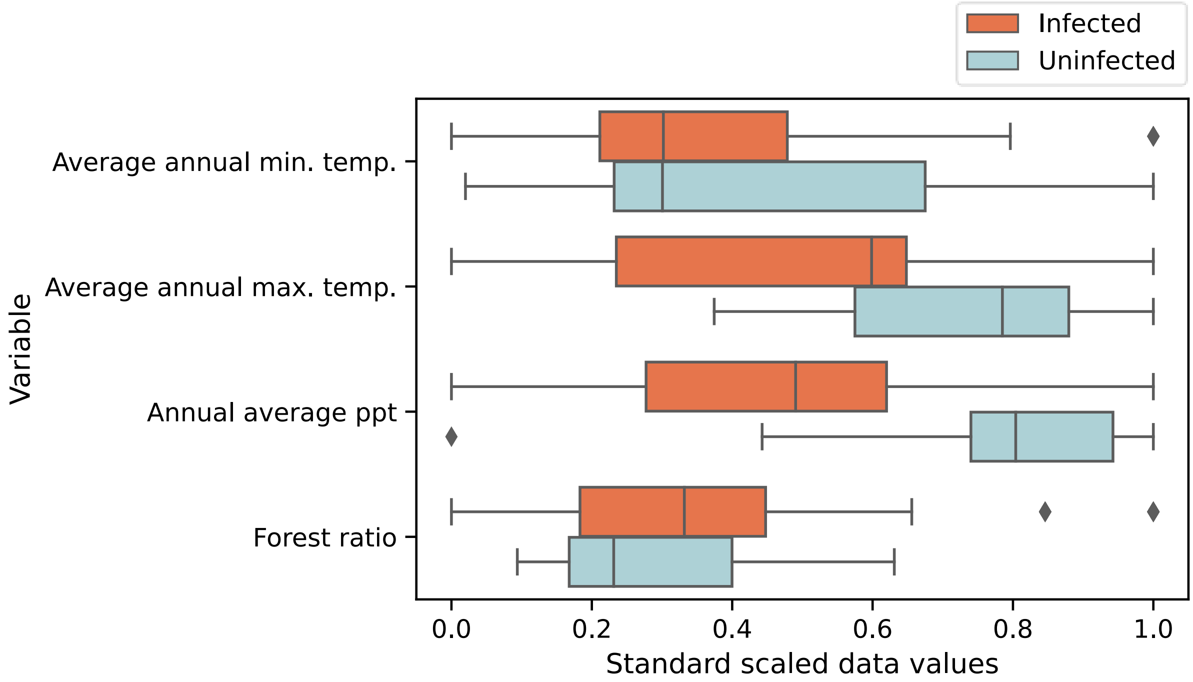

Supplementary Figure 4. Standardised and scaled environmental data comparing Uninfected (N=13) and Infected (N=242) by environmental variable. Source data can be found in Supplementary Table S1.

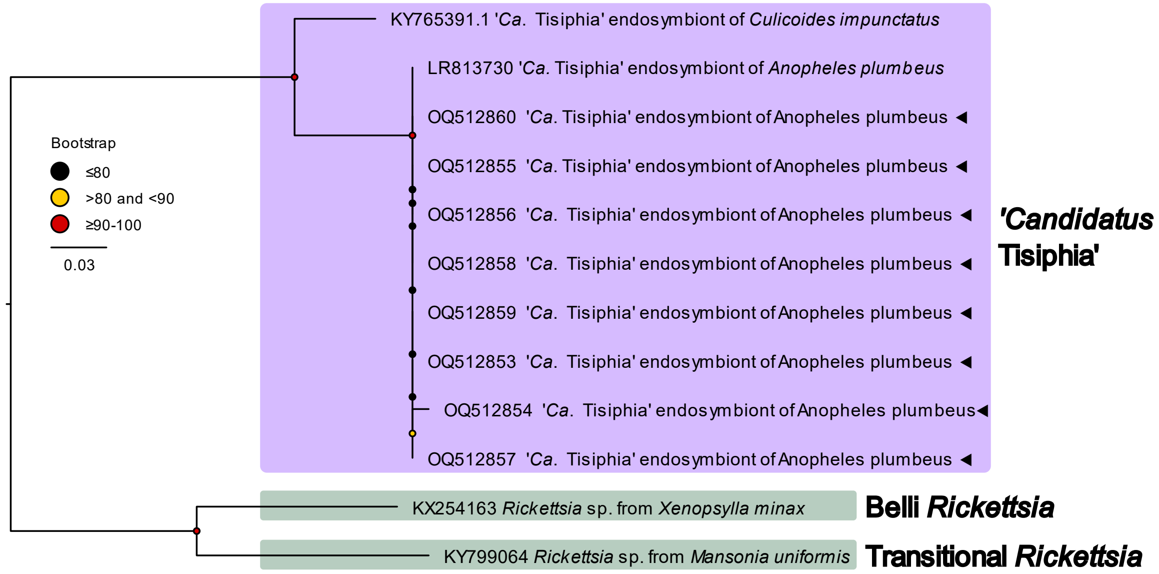

Supplementary Figure 5. Maximum likelihood tree for 17 kDa surface antigen (omp) for '*Candidatus* Tisiphia' extracted from *An. plumbeus*. Sequences from PCR screening are indicated by ◄ and bootstrap values based on 1000 replicates are indicated with coloured circles (red = 91-100, yellow = 81-90, black <= 80).

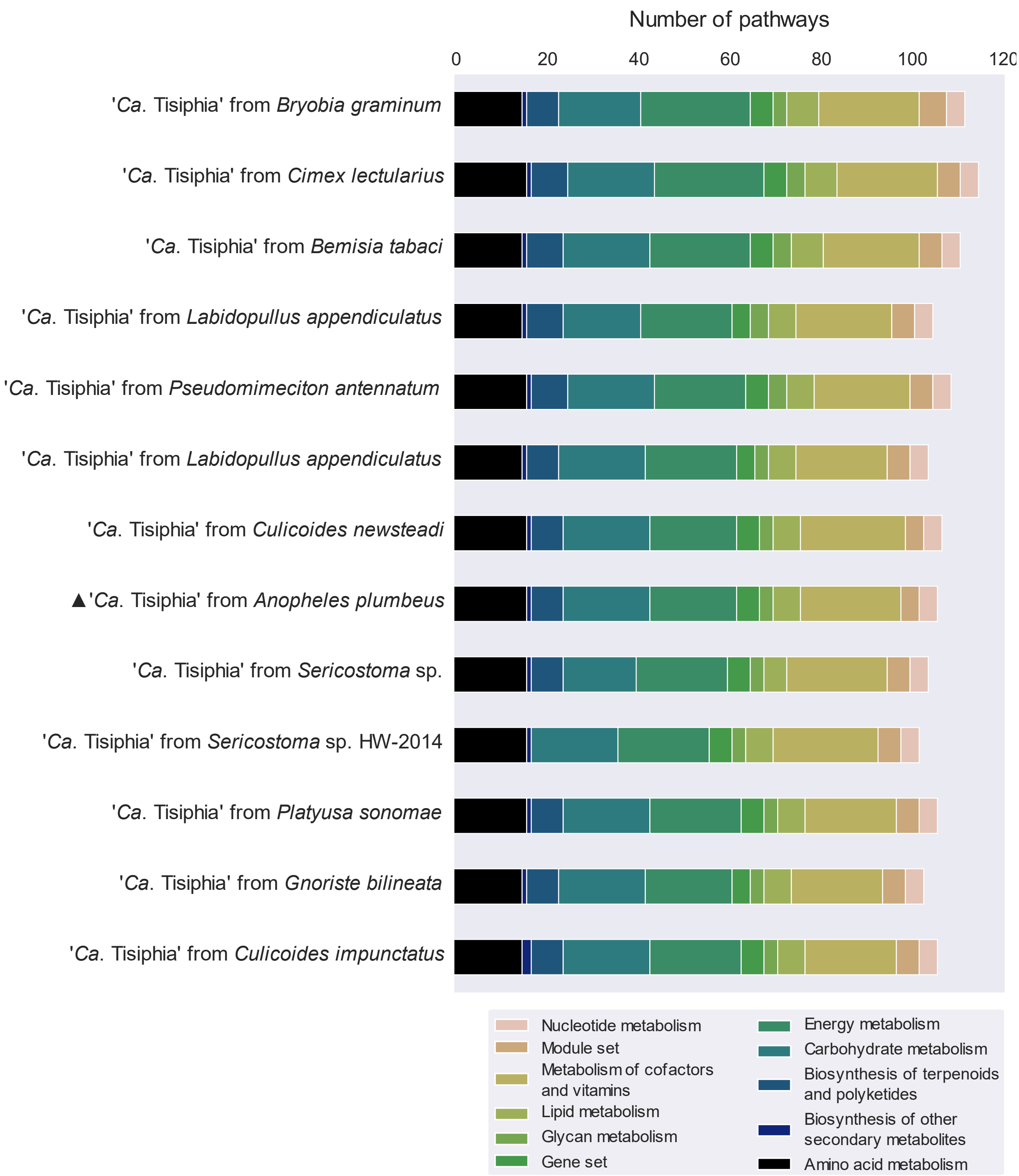

Supplementary Figure 6. KEGG module distribution in '*Ca*. Tisiphia'. The number of pathways found per genome annotated by KEGG module category for '*Ca*. Tisiphia'. Gene set and Module set are KEGG categories for signature modules that include various key functions like pathogenicity, metabolic capacity, and drug resistance. Lists of pathways and presence-absence data that make up each category can be found in Supplementary Tables S3 and S4. ▲ indicates the genome assembled in this study.

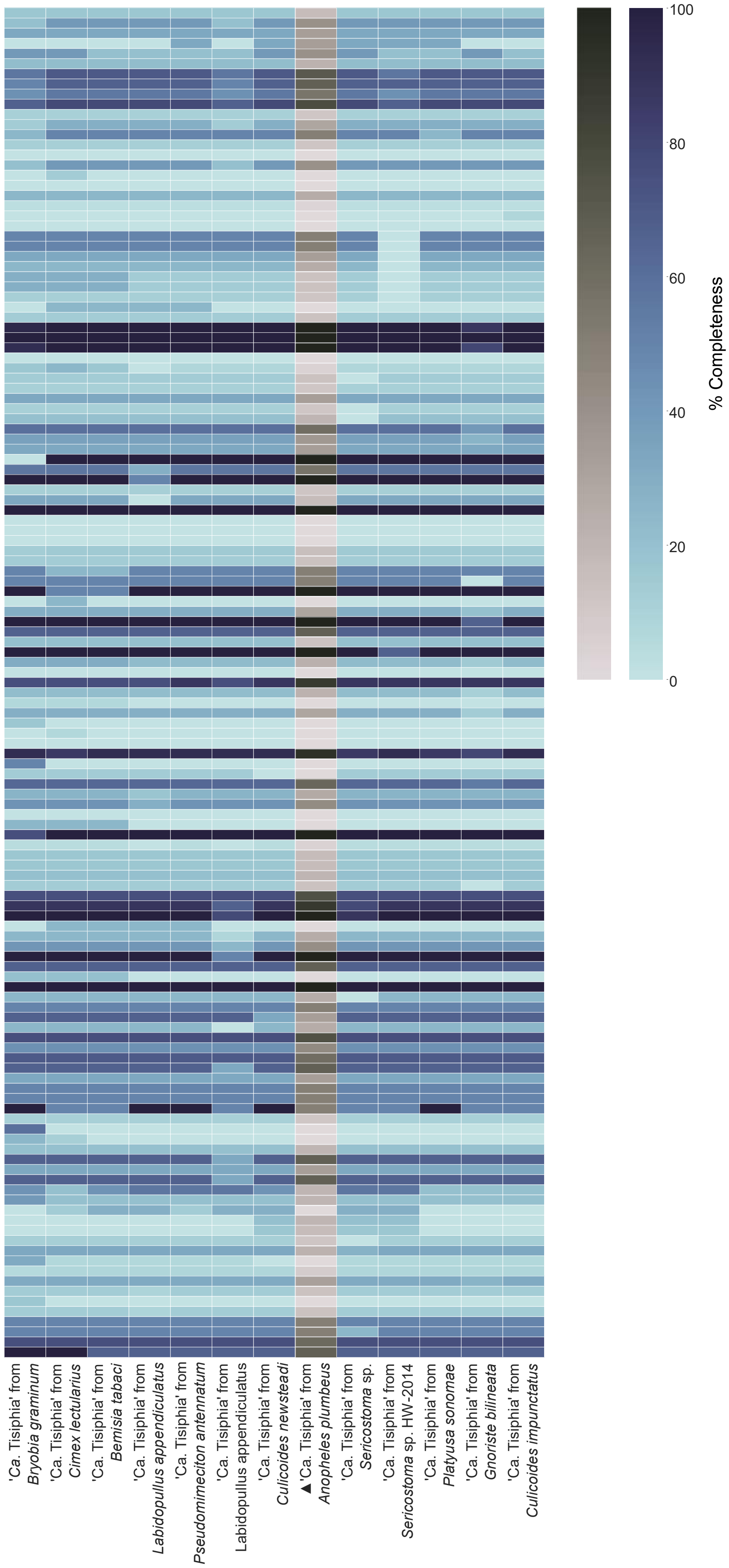

Supplementary Figure 7. Predicted completeness of KEGG kofam metabolic pathways across '*Ca.* Tisiphia'. The genome assembled in this chapter is coloured grey and indicated with ▲. Full metadata displaying the presence-absence data and pathway names can be found in Supplementary Tables S3 and S4.

Additional FISH results

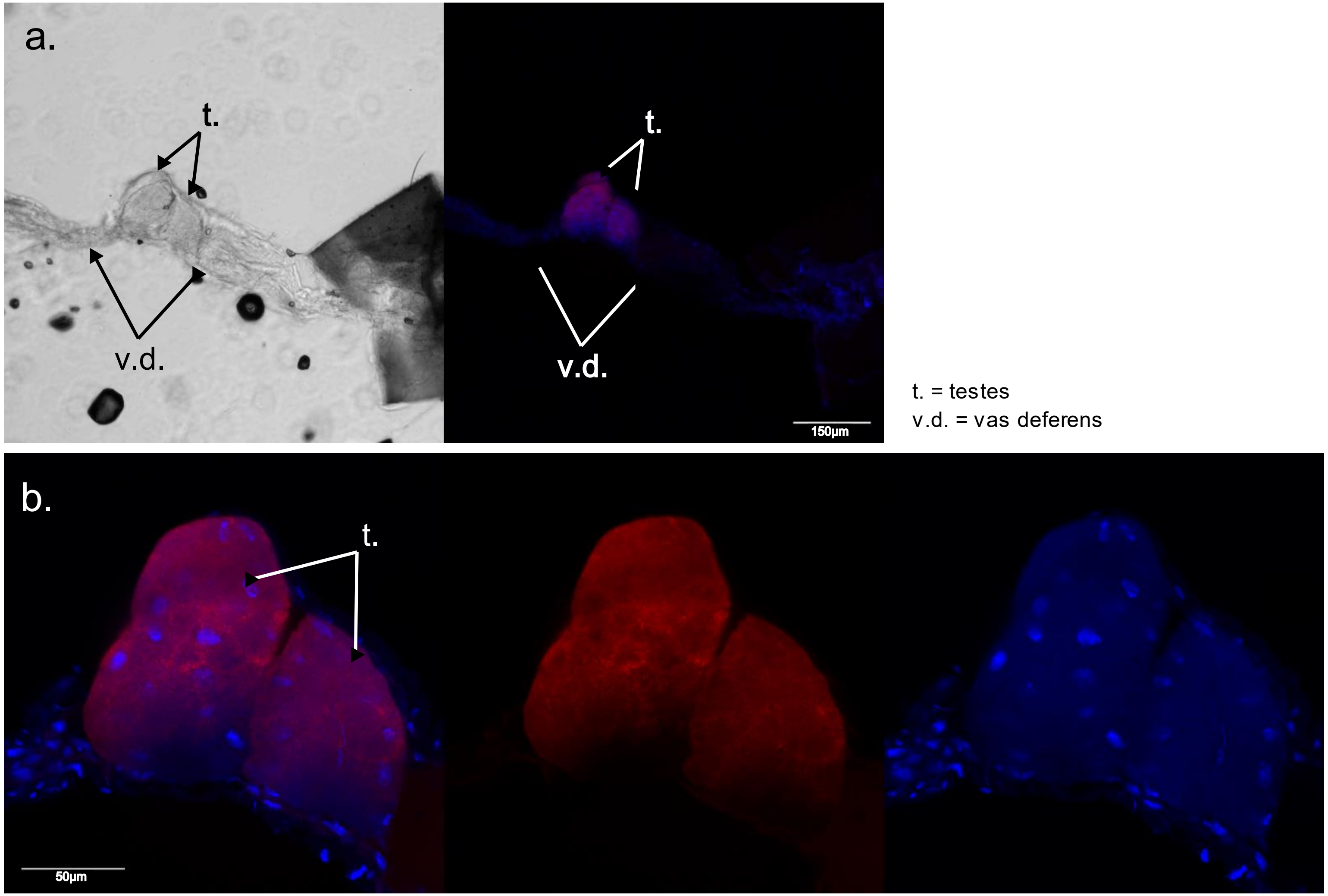

Supplementary Figure 8. Fluorescence in situ microscopy images of *Anopheles plumbeus* testes. Blue are host nuclei stained with Hoechst 33342, Red is ATTO-633 auto-fluorescence in the testes not '*Ca*. Tisiphia' staining. White bars indicate a) 150 micrometres and b) 50 micrometres.

References

Tsuchida, T. *et al.* (2002) 'Diversity and geographic distribution of secondary endosymbiotic bacteria in natural populations of the pea aphid, *Acyrthosiphon pisum*', *Molecular Ecology*, 11(10), pp. 2123–2135. Available at: https://doi.org/10.1046/j.1365-294X.2002.01606.x.
